# Management of Severe Bilateral Symptomatic Internal Carotid Artery Stenosis: Case Report and Literature Review

**DOI:** 10.3390/jpm14090893

**Published:** 2024-08-23

**Authors:** Mircea Robu, Bogdan Radulescu, Irina-Maria Margarint, Anca Dragan, Ovidiu Stiru, Gabriel-Petre Gorecki, Cristian Voica, Vlad Anton Iliescu, Horatiu Moldovan

**Affiliations:** 1 Faculty of Medicine, Carol Davila University of Medicine and Pharmacy, 050474 Bucharest, Romania; mircea.robu@drd.umfcd.ro (M.R.); ovidiu.stiru@umfcd.ro (O.S.); vladanton.iliescu@gmail.com (V.A.I.); horatiu.moldovan@umfcd.ro (H.M.); 2 1st Department of Cardiovascular Anesthesiology and Intensive Care, Prof. Dr. C. C. Iliescu Emergency Institute for Cardiovascular Diseases, 022328 Bucharest, Romania; anca.dragan1978.14@gmail.com; 3 Faculty of Medicine, Titu Maiorescu University, 031593 Bucharest, Romania; gabriel.gorecki@prof.utm.ro; 4 Department of Cardiovascular Surgery, Emergency Clinical Hospital Bucharest, 014461 Bucharest, Romania; cristian.voica@drd.umfcd.ro; 5 Academy of Romanian Scientists, 50711 Bucharest, Romania

**Keywords:** staged carotid artery endarterectomy, severe bilateral carotid artery stenosis, ischemic stroke

## Abstract

Multiple strategies for tandem severe carotid artery stenosis are reported: bilateral carotid artery endarterectomy (CEA), bilateral carotid artery stenting (CAS), and hybrid procedures (CEA and CAS). The management is controversial, considering the reported high risk of periprocedural stroke, hemodynamic distress, and cerebral hyperperfusion syndrome. We present the case of a 64-year-old patient with severe symptomatic bilateral internal carotid artery stenosis (95% stenosis on the left internal carotid artery with recent ipsilateral watershed anterior cerebral artery–medial cerebral artery (ACA-MCA) and medial cerebral artery–posterior cerebral artery (MCA-PCA) ischemic strokes and 90% stenosis on the right internal carotid artery with chronic ipsilateral frontal ischemic stroke) managed successfully with staged CEA within a 3-day interval. The patient had a history of coronary angioplasty and stenting. Strategies for brain protection included shunt placement after the evaluation of carotid stump pressure, internal carotid backflow, and near-infrared spectroscopy. A collagen and silver-coated polyester patch was used to complete the endarterectomy using a 6.0 polypropylene continuous suture in both instances. Management also included neurological consults after extubation, dual antiplatelet therapy, head CT between the two surgeries, myocardial ischemia monitoring, and general anesthesia. Staged CEA with a small time interval between surgeries can be an option to treat tandem symptomatic carotid artery stenosis in highly selected patients. The decision should be tailored according to the patient’s characteristics and should also be made by a cardiology specialist, a neurology specialist, and an anesthesia and intensive care physician.

## 1. Introduction

Carotid artery stenosis is responsible for 10% of ischemic strokes, one of the main causes of long-term disability. The prevalence of bilateral carotid artery stenosis is nearly 8–39% among patients with stroke [1,2]. The management of this situation is controversial and carries a high risk of periprocedural stroke, hemodynamic distress, and cerebral hyperperfusion syndrome [3]. Multiple strategies for tandem severe carotid artery stenosis are reported: bilateral carotid artery endarterectomy (CEA), bilateral carotid artery stenting (CAS), and hybrid procedures with CEA and CAS. However, there are no guidelines for the exact timing and order of these options. CEA and CAS can be performed simultaneously or staged. When considering the staged strategy, the optimal time interval between procedures is not well determined. With the hybrid approach, there is no consensus regarding which technique should be used first regardless of the staged or simultaneous strategy. Staged CEA was the first technique to emerge for tandem severe carotid artery stenosis and was considered for obvious reasons to be safer than simultaneous bilateral CEA [4,5]. Carotid artery stenting began as an alternative to this initial strategy, with the development of carotid artery stents, initially as an adjuvant to CEA, but also for high-risk patients [6] and patients with CEA restenosis [7]. We review different strategies and present the management of a 64-year-old male with severe bilateral symptomatic carotid artery stenosis, successfully managed with staged CEA with a 3-day interval.

## 2. Case Report

**Figure 1:**
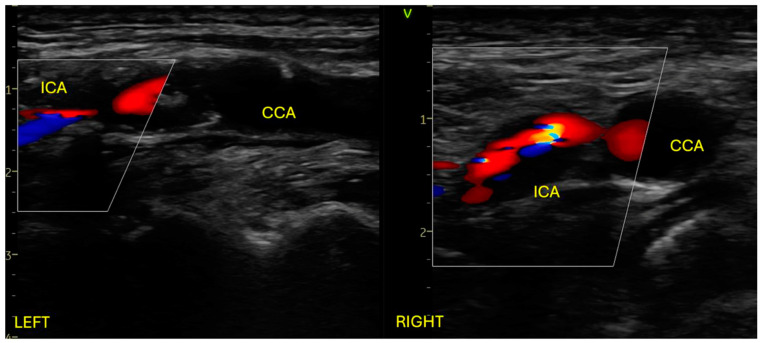
Arterial Doppler ultrasound of the carotid system showing bilateral severe stenosis at the level of common carotid artery bifurcation extending at the level of the internal carotid artery. ICA: internal carotid artery; CCA: common carotid artery.

We present the case of a 64-year-old male patient with a recent history (one month) of left ACA-MCA and MCA-PCA ischemic strokes with right hemiparesis, documented on cerebral CT. A chronic frontal right ischemic lesion was also documented. Neurological examination on admission revealed right minor central facial palsy, 3/5 MRC right hemiparesis, and mild right hemihypoesthesia, consistent with an NIHSS score of 4. Past medical history revealed ischemic heart disease, with severe stenosis at the proximal level of the anterior descending artery, treated with balloon angioplasty and two drug-eluting stents 6 months before admission. Also, the patient was diagnosed with hypertension, type II diabetes, and obesity. The patient was on dual antiplatelet therapy with Aspirin and Clopidogrel. At admission, he was stable, with a 110–120 mmHg systolic blood pressure, a 95 bpm regular pulse, and a 98% peripheral oxygen saturation. He had almost complete resolution of the right hemiparesis with the rest of the clinical examination within normal limits. The blood test revealed hyperlipidemia. The EKG showed normal sinus rhythm, without any ST-T changes, and the chest XR was without any pathological findings. Transthoracic echocardiography showed normal biventricular function, no significant valvular pathology, and no pericardial effusion. Arterial Doppler ultrasound of the carotid arteries revealed 90–95% stenosis at the level of the left common carotid artery bifurcation, extending to the internal carotid artery, and 85–90% stenosis at the level of the right common carotid artery bifurcation, extending at the level of the internal carotid artery. The atheromatous plaques responsible for the severe bilateral stenosis were both of mixt echogenicity (Figure 1).

**Figure 2:**
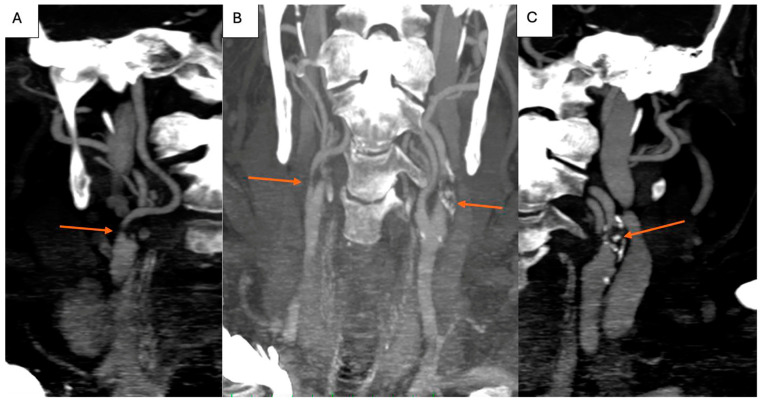
Cervical CT angiography showing right severe internal carotid artery stenosis ((**A**), arrow), left severe internal carotid artery stenosis ((**C**), arrow), and bilateral severe internal carotid artery stenosis ((**B**), arrows).

**Figure 3:**
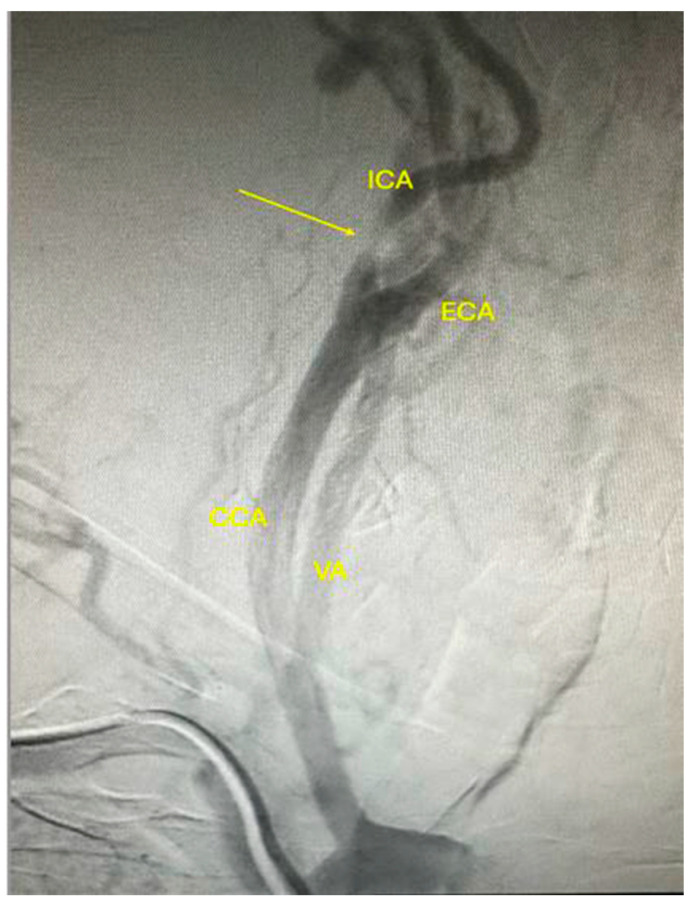
Carotid artery angiography showing suboclusive stenosis at the level of the left common and internal carotid artery with patent left vertebral artery. CCA: common carotid artery; ICA: internal carotid artery; ECA: external carotid artery; VA: vertebral artery.

Cervical CT angiography confirmed the severe lesions at the level of the carotid arteries (Figure 2A–C). Additionally, considering the history of ischemic heart disease, coronary angiography was obtained, and carotid angiography was performed in the same setting. Figure 3 shows severe suboclusive stenosis at the level of the left common and internal carotid artery. Unfortunately, the cannulation of the right carotid artery was not possible. Coronary arteries were without any significant stenosis, and the stents at the level of the anterior descending artery were patent.

The management strategy used was determined by a team that included a cardiology specialist, a neurology specialist, a vascular surgeon, an anesthesia specialist, and an intensive care physician. The decision was made to perform the left CEA first due to the recent ipsilateral ischemic stroke, and then as soon as possible afterwards the right CEA was performed. The reason for the relative short interval between surgeries included high-grade stenosis on the right internal carotid artery, with a history of ischemic stroke. Also, the mixed echogenicity aspect of the plaque at the echocardiography suggested a high risk of cerebral embolization. Stenting of the right carotid lesion as an alternative for right CEA was not an option for several reasons. First, our center’s experience with carotid stenting is low, with fewer than 10 cases per year, and second, from a technical point of view, we did not have stents with an embolic prevention system at that time. A head CT and a neurologic consultation were planned after the first intervention in order to identify hemorrhagic lesions that would contraindicate the second intervention. Also, the dual antiplatelet therapy was continued during the entire hospitalization, considering the high risk of cerebral thromboembolism. The patient signed both the informed consent and the publication consent form for the case and was scheduled for surgery the next day.

### 2.1. Surgical Technique

**Figure 4:**
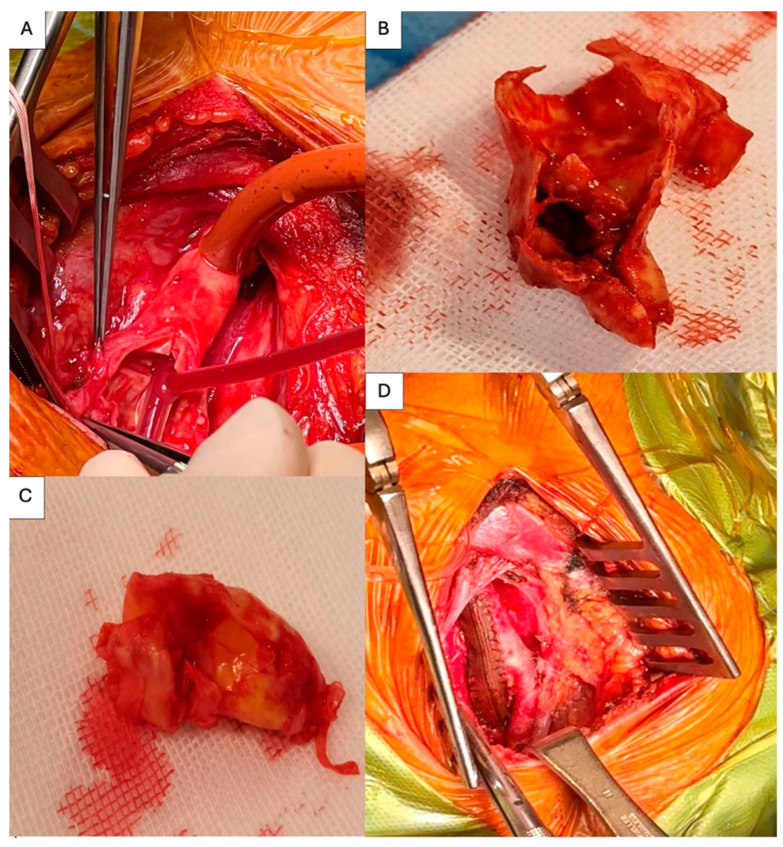
(**A**) A shunt inserted in the left common and internal carotid arteries. (**B**) Plaque extracted from the left carotid artery. (**C**) Plaque extracted from the right carotid artery. (**D**) Final result of left CEA with collagen and silver-coated polyester.

After the provision of general anesthesia, an incision was made along the left medial border of the sternocleidomastoid muscle. After the dissection of subcutaneous fat tissue and a platysma muscle incision, the sternocleidomastoid muscle was retracted laterally, the internal jugular vein was also retracted, and the carotid sheath was incised. The common, internal, and external carotid arteries were isolated carefully, so as not to injure the vagus and hypoglossal nerves. We administrated 3500 units of unfractionated heparin, and after 2 min, the carotid arteries could be clamped. The systolic blood pressure was maintained over 140 mmHg in order to augment cerebral blood flow during carotid artery clamping. Retrograde pressure at the level of the internal carotid artery was measured (24 mmHg) and was below 40 mmHg. A longitudinal incision was made, beginning at the level of the common carotid artery, moving over the carotid artery bifurcation, and extending to the level of the internal carotid artery 1 cm distal to the end of the plaque felt at palpation. The clamp at the level of the ICA was removed, and backflow was evaluated and deemed unsatisfactory. We used near-infrared spectroscopy (NIRS) to monitor cerebral oxygenation, and the values dropped on the left side after carotid clamping from 65% to 45%. All of the above (reduced backflow, reduced retrograde blood pressure, drop in NIRS) led us to insert a shunt in order to augment cerebral perfusion during carotid clamping (Figure 4A). After the shunt insertion, NIRS values returned to normal. Continuous transcranial Doppler ultrasonography and EEG monitoring were not available in our institution. The plaque was excised down to the level of the intima, and two 7.0 polypropylene sutures were used to fix the layers at the distal end of the endarterectomy. Macroscopic examination of the plaque confirmed the mixed heterogenicity shown on the Doppler ultrasound with thrombotic material attached to the inner surface (Figure 4B). Figure 4C shows the plaque extracted at the time of the right CEA. A collagen and silver-coated polyester patch was used to complete the endarterectomy using a 6.0 polypropylene continuous suture. After the shunt was extracted, the arteries were deaired, the suture was finished, and then all arteries were declamped in the reverse order of clamping: the external carotid artery, common carotid arteries, and internal carotid artery. The carotid artery clamp time was 24 min. The final result is shown in Figure 4D. A surgical drain was inserted via contra incision. Both CEAs were performed with the same surgical technique. The second CEA had a 21 min period of carotid artery clamping. For the second CEA, shunt insertion was not necessary.

### 2.2. Postoperative Evolution

After the left CEA, the patient was extubated after 3 h. He had no new neurological deficits according to the neurological consult. Myocardial enzymes, serial EKG, and transthoracic echocardiography showed no signs of myocardial ischemia in the immediate postoperative period. The only postoperative complication was postoperative arterial hypertension. Regarding blood pressure values, the highest value was for systolic blood pressure between 170 and 190 mmHg when the sedation was stopped. It was rapidly managed with intravenous calcium channel blockers (Nicardipine) and continued the whole stay in intensive care with normal values for the blood pressure. The patient was transferred to the surgical ward the following day with oral medication for hypertension. The surgical drain was extracted on the same day. On the second day, we obtained a head CT and a neurological consult. Because there were not any clinical or imaging signs of new cerebral ischemia or cerebral hemorrhage, the neurologist cleared the patient for the second CEA. On the third postoperative day, the patient was transferred to the operating room, and a right CEA was performed using the same technique described above. Postoperative evolution was uneventful, the patient was extubated after 5 h, no neurological complications were documented, transthoracic echocardiography was performed within normal limits, and myocardial enzymes and serial EKG showed no signs of myocardial ischemia. He was transferred to the surgical ward the following day. The surgical drain was extracted on the second day after the right CEA, and no cervical hematoma was observed at daily evaluations. After another neurological consultation and a final cardiological examination, the patient was discharged on the seventh day after the initial left CEA. At discharge, hypoesthesia and hemiparesis were almost completely reversed with mild deficit persistence on right upper limb, consistent with an NIHSS score of 2 (minor facial palsy and mild deficit on the right upper limb). Follow-up at 1, 3, and 6 months and 1 year were uneventful.

## 3. Review

Stroke is the second most common cause of death worldwide and is characterized by high morbidity. Furthermore, half of stroke survivors have severe disability, and subsequently, these patients are a heavy burden on public health [8]. Recurrent ischemic stroke contributes massively to this burden and inflicts potentially debilitating sequelae on a group of already fragile patients. Thus, preventive measures should be developed and implemented. While the control of risk factors is beneficial for secondary prevention, the identification of the specific mechanism, such as internal carotid artery stenosis ipsilateral to ischemic stroke or transitory ischemic accident (TIA), will guide decision-making for further prevention [9].

Reported for the first time in 1954 by Eastcott et al. [10], carotid endarterectomy (CE) has proven to have a beneficial effect on stroke prevention in patients with internal carotid artery stenosis. In 1991, the North American Symptomatic Carotid Endarterectomy Trial (NASCET) and the European Carotid Surgery Trail (ECST) reported a beneficial effect of CE in patients with angiographic evidence of symptomatic stenosis with comparable rates of periprocedural rates of stroke and death. They concluded that CE was highly beneficial for symptomatic stenosis, ranging from 70% to 99%, and moderately beneficial for symptomatic carotid stenosis, ranging from 50 to 69% [11,12].

Since then, numerous societies have elaborated guidelines for the management of extracranial carotid stenosis. The 2021 American Heart Association Guidelines for the Prevention of Stroke in Patients with Stroke and Transient Ischemic Attack (TIA) recommend CE for both 70% to 99% and 50% to 69% carotid artery stenosis and ipsilateral TIA or nondisabling ischemic stroke occurring within the past 6 months if the perioperative morbidity and mortality risk is below 6%. For the 50% to 69% group of patients age, sex and comorbidities should be considered before surgery. CE is also the preferred method for stroke prevention over carotid artery stenting (CAS) in patients over 70 years old, while CAS is recommended in severe carotid artery stenosis (over 70%) with a high risk of surgery [13]. The European Society for Vascular Surgery Clinical Practice Guidelines on the Management of Atherosclerotic Carotid and Vertebral Artery Disease have recommendations for both symptomatic and asymptomatic patients [14]. For 60% to 99% asymptomatic carotid stenosis cases, CE is recommended in the presence of one or more imaging or clinical characteristics that may be associated with increased risk of stroke, provided that 30-day stroke/death rates are below 3% and patient life expectancy exceeds five years. CAS is recommended if surgery is deemed high risk in the same conditions. Symptomatic patients treated within the preceding six months with a 70–99% carotid artery stenosis have a class I indication for CE provided that the risk of 30-day risk of death/stroke is below 6%. Patients with 50–69% carotid artery stenosis have a class IIa indication. CAS is the preferred method for treatment in patients over 70 years old in accordance with the American Heart Association Guidelines. CE should be performed within 14 days from the onset of symptoms to increase the likelihood of a stroke-free outcome, a recommendation stated in both of the societies’ guidelines. The European Stroke Organization recommends CE for patients with more than 60% asymptomatic carotid artery stenosis with an increased risk of stroke with medical therapy alone, and this is also a recommendation for patients over 75 years old with a life expectancy of more than 5 years. Patients with symptomatic 70–99% carotid artery stenosis have a strong recommendation for CE, while CE is suggested for symptomatic 50–69% stenosis. Early CE within 2 weeks of the first neurological event also has a strong recommendation [15].

While it is clear that symptomatic severe carotid artery stenosis has strong recommendation for CE, the guidelines do not specify the management for severe bilateral carotid artery stenosis, especially in the presence of symptoms and or ulcerous high-risk embolism plaques. The frequency of bilateral carotid stenosis varies and is reported to be between 3.2% and 39% in the literature [2,16,17]. In this group of patients, impaired cerebral hemodynamics may contribute to cognitive dysfunction, neurological symptoms, and even stroke, even in the absence of symptoms, and it is believed that this group of patients could benefit from the more aggressive management of carotid revascularization [18]. The management of this situation carries a high risk of periprocedural stroke, besides hemodynamic complications and hyperperfusion syndrome [17]. Strategies for the treatment of these patients include bilateral CEA, bilateral carotid artery stenting (CAS), and a hybrid approach with CEA and CAS. However, the optimal sequence that is simultaneous or staged has not been determined.

### 3.1. Bilateral Carotid Endarterectomy (BCAE)

Bilateral CEA can be performed simultaneously (sbCEA) or staged (SCEA) with a variable interval between the two surgeries. Advocates of SCAE consider that leaving an interval of time between the two interventions reduces the risk of brain injury, but the optimal interval between the first and second surgery has not been defined [19,20]. Kim et al. performed SCAE in 43 patients within 30 days or less (the mean time interval was 12 days). Compared with the group of patients with unilateral CEA, there were no differences with regard to nerve cranial nerve palsy and non-neurological CEA-related complications. Age was the only risk factor associated with ipsilateral stroke in multivariate analysis, while SCEA was not associated with stroke. The two groups did not differ significantly with regard to stroke, myocardial infarction, or death during the periprocedural period or ipsilateral stroke within 3 years after CEA. Also, similar rates of survival were communicated [20]. Rodrigues et al. performed SCEA with a main interval of 4 days in 154 patients and reported no increase in immediate and 30-day morbidity and mortality when compared to unilateral CEA [21]. Darling et al. report 102 cases with a mean interval between surgeries of 2 days with no operative mortality and only one permanent neurological deficit. They communicate a stroke mortality rate of 1% and a 1.5% rate of transient neurological deficits comparable with those undergoing unilateral CEA [22]. Schroeder et al. reported in a series of 56 patients that postendarterectomy hypertension was significantly higher following the second procedure when the procedures were performed less than 3 weeks apart and was linked with the occurrence of new neurological symptoms. The author advises at least a three-week interval between the first and the second CEA and a conservative attitude towards the contralateral asymptomatic lesion [5]. In accordance with Schroeder, several other reports describe the association between postoperative hypertension and new-onset neurological symptoms, supporting the idea that the second CEA should be performed after more than 3 weeks [23,24,25]. A short interval time between CEA and the occurrence of postoperative hypertension was also reported by Maxwell et al. He communicated a 5.6% death and stroke rate in 161 patients with SCEA [26]. The most recent study to this date investigating changes in blood pressure after CEA identified hypertension as an independent predictor of perioperative stroke following bilateral CEA irrespective of the interval between surgeries. Also, bilateral CEA was associated with postoperative hypertension together with gender, emergency admissions, and plaque morphology [27].

Those in favor of SBCEA advocate that repeated anesthesia, repeated surgical stress, prolonged intensive care and hospitalization times, and increased risk of cranial hemorrhage because of second heparinization increase the risk of SBCEA [28]. Although neither SCEA nor SBCEA have been proven to be the correct strategy for bilateral severe carotid artery stenosis, particular lesions like patients with bilateral stenosis of more than 90% and those with ulcerous, calcified, and high-risk embolism plague SBCEA seem to be a rationale choice [28,29,30].

Ketonen et al. reported, in 1979, a series of 18 patients with SBCEA. He communicated a 3.8% hospital mortality, 5% rate of transient neurological deficits, and 5% rate of permanent neurological deficit [4]. While the results of the above study do not favor SBCEA, multiple studies comparing SCEA with SBCEA report no significant differences between the two aspects. Farask et al. compared 6 patients with SBCEA with 20 patients with SCEA and found no differences regarding mortality, myocardial infarction, respiratory problems, or permanent neurological damage of the central nervous system. The only complication reported in the SBCEA was transient hypoglossal paresis [29]. Dimakakos et al. report their initial experience, comparing 11 BSCEA with 12 SCEA and 155 cases of unilateral CEA regarding death, myocardial infarction, or permanent neurological dysfunction. No differences were found, and reports in the SBCEA group included a case of transient ischemic attack and transient vocal cord paresis [31]. The same author reported the same results, with no significant differences between 17 BSCEA and 17 SCEA with a zero mortality rate. The conclusion favors SCEA, with SBCEA being reserved for strictly selected patients [29].

Besides an experienced surgical team, the type of anesthesia appears to play an important role when considering SBCEA. Regional anesthesia offers the advantage of monitoring the neurological status and determining the shunt indication, while general anesthesia seems to have a protective effect for cerebral hypoxia [32]. Regional anesthesia also decreases the need for shunt placement from 43% to 14%, a significant advantage considering that shunt placement has a 1% to 3% risk of embolism and dissection [30,31]. Also, general anesthesia is linked to a study with a higher incidence of myocardial infarction [33]. Despite the fact that regional anesthesia does not appear to influence the postoperative results of CEA and has the advantage of increasing patient comfort, no need for recovery like general anesthesia and reducing hospitalization costs and complications like phrenic nerve, vagus nerve, and stellate ganglion lesions can be observed [28].

### 3.2. Hybrid Procedures and Bilateral Carotid Artery Stenting

Another strategy for bilateral severe carotid artery stenosis is a hybrid approach with carotid artery stenting (CAS) and either staged or simultaneous CEA.

Xu et al. report a series of eight patients with severe bilateral carotid stenosis with simultaneous CAS and CEA. Three patients had CAS before CEA and five patients had CEA as the first intervention. There were no deaths, and only one patient developed hyperperfusion syndrome [34]. Hokari et al. discuss two patients with severe bilateral carotid artery stenosis with CEA performed first for the symptomatic side and CAS performed for the contralateral side after three weeks. There were no deaths, one of the patients developed hyperperfusion syndrome after CEA with full recovery and the second patient developed severe hypotension requiring catecholamines but also with good evolution [34]. Beach et al. also report 22 patients with staged CEA and CAS. CEA was performed first with a 4.5% rate of perioperative stroke and myocardial infarction and a 9.1% rate of cranial nerve injuries [35].

Shchehlov et al. report 10 years of experience with bilateral simultaneous CAS in 39 patients. In all cases, stenting was performed with success, with two periprocedural neurological complications, one TIA, and one minor stroke, as well as with no deaths or myocardial infarction recorded [1]. Chen et al. also report 10 patients with simultaneous bilateral CAS deemed high risk for surgery, and Mubarak et al. report 5 cases treated for CEA restenosis, with no periprocedural complications [6,7]. Lai et al. performed a meta-analysis to evaluate the safety of bilateral simultaneous CAS. Based on 333 cases reported in the literature, they conclude that besides hyperperfusion syndrome, other periprocedural complications including hemodynamic depression, stroke, and myocardial infarction are comparable with unilateral CAS [3]. Oshita et al. compare simultaneous with staged bilateral CAS, and their conclusion is that bilateral CAS performed simultaneously was not inferior to staged CAS, with an obvious advantage when considering the length of hospital stay [36]. Based on the current data, simultaneous bilateral CAS might increase the risk of intra- or postoperative bradycardia and/or hypotension due to bilateral baroreceptor stimulation or hyperperfusion syndrome resulting from an increase in bilateral intracranial blood flow. On the other hand, simultaneous BCAS has the advantage of being less expensive and more convenient for patients than staged BCAS.

All authors (Table 1) conclude that patients with tandem severe carotid artery stenosis should be carefully examined and the best treatment strategy should be assessed using a multidisciplinary approach.

## 4. Discussion

We presented a case of a 64-year-old male patient with severe bilateral symptomatic carotid artery stenosis. He was successfully managed with staged CEA with a 3-day interval between surgeries. While many strategies are reported for this condition (staged CEA, simultaneous bilateral CEA, hybrid procedure), there are no guidelines and no strategy has been proven superior. We consider that a patient-tailored strategy should be planned considering patient comorbidities and the risk of cerebral thromboembolism. In our case, the presence of severe symptomatic bilateral carotid artery stenosis (recent left ischemic stroke with right hemiparesis and chronic ischemic frontal lesion on the right side), with mixed echogenicity and high risk of cerebral embolism, led us to perform carotid endarterectomy first on the left side and then right carotid endarterectomy within a three-day interval. This strategy is supported by data from the literature. Both Farsk et al. [30] and Dimakakos et al. [31] reported no difference between simultaneous and staged CEA. Rodriguez et al. [22] observed no increase in immediate and 30-day morbidity and mortality when they compared 154 staged CEA cases with a mean interval of 4 days with unilateral CEA. Also, Darling et al. [23] reported a low (1%) combined stroke and mortality rate after analyzing 102 staged CEA with a mean interval of 2 days between surgeries, while Kim et al. [21] did not find any association with ipsilateral stroke in 43 staged CEA cases. However, Maxwell et al. [27] reported a higher rate of stroke and death (5.6%) than Darling et al. A possible explication for this difference could be the evolution of brain protection strategies. We did not proceed with simultaneous bilateral CEA because of the risk of hyperperfusion syndrome associated with severe bilateral stenosis especially in our case with more than 90% bilateral stenosis. The patient developed postoperative hypertension after the first left CEA in accordance with several reports that link this complication to a less than 3-week interval between surgeries [5,28]. This complication was rapidly managed with intravenous calcium channel blockers and did not affect the evolution of the patient. Also, considering the history of ischemic cardiomyopathy with stent implantation, we considered that longer operation times associated with simultaneous bilateral CEA could increase the risk of myocardial ischemia complications. Also, another reason in favor of staged CEA was the high risk of bleeding and cervical hematoma occurrence associated with the dual antiplatelet therapy if not discontinued.

While bilateral CAS is reported to be a safe alternative for CEA with studies that report no complications [6,7,27], it was not an option in our case even if we consider only CAS of the right internal carotid artery. The reason was both the lack of experience and materials (embolic prevention system) needed for a safe procedure.

The planned management also included the following: dual antiplatelet therapy not discontinued because of the risk of thromboembolism; a neurological consultation immediately after each extubation; myocardial ischemia monitoring with serial EKG, myocardial enzymes, transthoracic echocardiography and a coronary angiography performed in the preoperative setting to evaluate the stents implanted and other possible coronary stenosis; a neurological consultation and a head CT before the second CEA in order to evaluate the safety of performing surgery (we looked for new imaging or clinical signs of cerebral hemorrhage that would contraindicate the second CEA); and general anesthesia, with a neurological and cardiological consultation before discharge.

Considering the surgical technique used, strategies for brain protection during carotid artery clamping are essential in this case of severe bilateral carotid artery stenosis. While continuous transcranial Doppler ultrasonography and EEG monitoring were not available at our institution, we measured the pressure in the internal carotid artery after clamping (carotid stump pressure), and we evaluated the backflow in the internal carotid artery. Also, NIRS was used to monitor cerebral oxygenation. A carotid pressure stump pressure below 40 mmHg [38,39], an unsatisfactory backflow, and a more than 20% reduction from baseline in cerebral oxygenation led us to proceed with shunt insertion. Also, careful hemostasis and surgical drains were essential for reducing the risk of cervical hematoma.

General anesthesia was used because studies report a protective effect for cerebral hypoxia. Also, the type of anesthesia does not seem to influence the postoperative results of CEA. Even though regional anesthesia reduces the need for shunt placement, in our case, it was obvious that shunt placement would be very likely. We also wanted to avoid specific neurological complications associated with this type of anesthesia like phrenic nerve, vagus nerve, and stellate ganglion lesions. To reduce the risk of myocardial infarction associated with general anesthesia, we chose to do a staged procedure to minimize the operative times, and dual antiplatelet therapy was not discontinued considering the history of coronary artery stenting 6 months prior.

## 5. Conclusions

The management of tandem severe symptomatic carotid artery stenosis can be challenging considering the high risk of periprocedural stroke and the lack of consensus regarding this situation. Staged CEA with a small time interval between CEAs can be an option in patients with a high risk of cerebral thromboembolism when postponing the second CEA for another hospital admission. Although simultaneous bilateral CEA is reported, the choice of surgical and anesthesia strategy should be tailored to each patient. Considering the favodrable evolution of this case, we consider staged CEA for bilateral severe symptomatic (more than 90%) carotid artery stenosis to be a safe strategy in very selected cases.

## Figures and Tables

**Table 1:** Studies that report the management of severe bilateral carotid artery stenosis. CEA: carotid artery endarterectomy; CAS: carotid artery stenting.

Autor	Year	Management	Complications
Xu et al. [34]	2016	8 simultaneous CEA and CAS	Hyperperfusion syndrome
Kim et al. [21]	2015	43 staged CEA within 30 days	No association with ipsilateral stroke
Farask et al. [30]	2001	12 bilateral simultaneous CEA com pared to 20 stagedCEA	No major neurological complications; no difference in the gropus
Dimakakos et al. [31]	1996	12 bilateral simultaneous CEA and 22 staged CEA	No differences between gropus; one case of TIA and another case of transient vocal cord paresis in the bilateral simultaneous CEA group
Darling et al. [23]	1996	102 staged CEA within 2 days	Combined stroke mortality rate of 1%
Hokari et al. [3]	2014	Hybrid procedure: 2 CEA (symptomatic side) and CAS after 2 months	Severe hypotension after CAS
Schroeder et al. [5]	1986	56 staged CEA within 3 weeks	Postendarterectomy hypertension was significantly higher following the second procedure, when operations were staged less than 3 weeks apart
Maxwell et al. [27]	1992	161 staged CEA	5.6% stroke and death rate; death was associated with postoperative hypertension and a short intersurgical interval
Kavakli et al. [29]	2015	1 bilateral simultaneous CEA with regional anesthesia	No complications
Ketonen et al. [4]	1979	18 bilateral simultaneous CEA	3.8% mortality, 5% permanent neurological deficits
Mubarak et al. [7]	1998	5 bilateral CAS	No complications
Chen et al. [6]	2004	10 bilateral CAS	No complications
Sultan et al. [28]	2024	91 staged CEA	Postoperative hypertension as a crucial independent predictor of perioperative stroke
Jumpei et al. [37]	2019	8 bilateral simultaneous CAS and 4 staged bilateral CAS	Bilateral simultaneous CAS is not inferior to staged CAS
Beach et al. [36]	2019	22 hybrid procedures: staged CEA and CAS	One perioperative stroke (4.5%), one patient with postoperative myocardial infarction (4.5%), and two patients (9.1%) with cranial nerve injuries
Shchehlov et al. [1]	2022	39 bilateral simultaneous CAS	Two periprocedural neurological complications, one transient ischemic attack, and one minor stroke
Lai et al. [3]	2019	333 bilateral simultaneous CAS	Except for hyperperfusion syndrome, all other periprocedural complications including hemodynamic depression, stroke, and MI were comparable with the literature reports on unilateral carotid artery stenting
Rodriguez et al. [22]	2001	154 staged CEA (4 days)	No increase in immediate and 30-day morbidity and mortality when compared to unilateral CEA

## Data Availability

Data available on request.
